# Aeration Alleviated the Adverse Effects of Nitrogen Topdressing Reduction on Tomato Root Vigor, Photosynthetic Performance, and Fruit Development

**DOI:** 10.3390/plants13101378

**Published:** 2024-05-15

**Authors:** Jingang Li, Pingru He, Qiu Jin, Jing Chen, Dan Chen, Xiaoping Dai, Siyu Ding, Linlin Chu

**Affiliations:** 1 College of Agricultural Science and Engineering, Hohai University, Nanjing 211100, China; lijingang68@sina.com (J.L.);; 2 State Key Laboratory of Hydrology-Water Resources and Hydraulic Engineering, Nanjing Hydraulic Research Institute, Nanjing 210029, China

**Keywords:** underground drip irrigation, micro-nano bubble, root vigor, dry matter accumulation, nitrogen accumulation

## Abstract

To explore the compensation effect of aeration on tomato vegetative and reproductive growth in arid and semi-arid areas, a two-year field experiment was conducted with four micro-nano aeration ratios (0%, 5%, 10%, and 15%) and three nitrogen topdressing levels (80, 60, and 40 kg·ha^−1^) during the tomato growth period in Ningxia, China. The results showed that increasing the aeration ratio in the range of 0–15% was conducive to the enhancement of tomato root vigor (the ability of triphenyltetrazolium chloride to be reduced, 3–104%) and the leaf net photosynthetic rate (14–63%), favorable to the facilitation of plant dry matter accumulation (3–59%) and plant nitrogen accumulation (2–70%), and beneficial to the improvement of tomato yield (12–44%) and fruit quality. Interestingly, since the aeration ratio exceeded 10%, the increase in the aeration ratio showed no significant effects on the single-fruit weight, tomato yield, and fruit quality. Moreover, with aerated underground drip irrigation, properly reducing the traditional nitrogen topdressing level (80 kg·ha^−1^) by 25% was favorable for enhancing tomato root vigor (5–31%), increasing tomato yield (0.5–9%), and improving fruit soluble solid accumulation (2–5%) and soluble sugar formation (4–9%). Importantly, increasing the aeration ratio by 5% could compensate for the adverse effects of reducing the nitrogen topdressing level by 25% by improving the leaf photosynthetic rate, promoting plant dry matter accumulation, increasing tomato yield, and enhancing the soluble solid and soluble sugar accumulation in tomato fruits. Synthetically considering the decrease in the nitrogen topdressing amount, leading to plant growth promotion, a tomato yield increase, and fruit quality improvement, a favorable nitrogen topdressing level of 60 kg·ha^−1^ and the corresponding proper aeration ratio of 10% were suggested for tomato underground drip irrigation in the Yinbei Irrigation District of Ningxia.

## 1. Introduction

Both cultivated land degradation and non-point source pollution have gradually become serious restrictions for agricultural sustainable development in arid and semi-arid regions, posing serious challenges for regional crop production. In addition to specific geographic conditions, complicated climatic factors, intensive agricultural activities, etc. [1], unreasonable irrigation modes and over-fertilization can also result in the decline of farmland soil productivity [2]. Thus, highly efficient water-saving irrigation technologies and scientific fertilization systems are urgently required for sustainable crop production in arid and semi-arid agricultural regions.

As one of the high-efficiency irrigation technologies, aerated underground drip irrigation is conducted by transferring water-gas mixture through underground drip irrigation systems to increase soil moisture and improve soil oxygen content in the crop rootzone [3,4,5]. Compared with the traditional surface drip irrigation technologies, aerated underground drip irrigation provides sufficient oxygen for a series of physiological actions of plant roots and the phytoedaphon [4], which usually contribute to the improvement of soil nutrient use efficiency [2,6,7,8,9] and the rootzone soil microenvironment [10,11], an increase in soil fertility [10,12] and crop production [8,9], and the facilitation of plant growth and fruit/grain development. Additionally, the mixing of water, fertilizer, and air both in time and space can be achieved through underground drip irrigation systems [13,14], which is more favorable for quick-acting nutrient release in the soil [7,10,15], the efficiency improvement of farmland fertilizer use [7], and crop yield increases. The current studies about aerated underground drip irrigation mainly focus on the influences of aeration proportions on crop growth characteristics and soil physical–chemical properties. However, few studies have paid attention to the effects of aeration ratios on root vigor, leaf physiological performance, and tomato fruit development with underground drip irrigation technology.

Tomato, a cash crop sensitive to soil oxygen stress, is widely cultivated in arid and semi-arid areas [16]. However, after long-term tomato cultivation, farmland tends to experience problems (compaction, acidification, salinization, etc.) due to intensive cultivation, excessive fertilization, and improper irrigation [4,17]. Soil degradation is generally accompanied by a reduction in soil oxygen content and permeability [7,18], the inhabitation of water and nutrient absorption by tomato roots [18], and irreversible damage to tomato fruits [19,20]. Extensive studies have indicated that aeration irrigation is conducive to increasing the soil oxygen content in the rhizosphere of tomato, alleviating the damage resulting from hypoxic stress and salt stress in tomato plants [8,21], enhancing the normal physiological activities of tomato plants [22], accelerating tomato growth [7,8,16], and increasing tomato yield [20,23].

To reduce farmland non-point source pollution, decrease field greenhouse gas emissions, and improve fertilizer use efficiency, the inefficient traditional fertilization modes with high fertilizer application levels for high agricultural production are no longer applicable. However, crop yields usually show a decreasing trend with the decline in the fertilizer application amount. Thus, effective measures (such as oxyfertigation) are necessary to alleviate the detrimental effects of fertilization reduction for considerable yield. Few studies have concentrated on the compensatory influence of aeration for fertilization reduction on plant growth and development, which is important for high-efficiency irrigation and precise fertilization in arid and semi-arid areas. Thus, a two-year field experiment with four micro-nano aeration ratios and three nitrogen topdressing levels was conducted in the Yinbei Irrigation area of Ningxia, China, with the following aims: (1) to investigate the coupling effects of the aeration ratio and nitrogen topdressing level on tomato root vigor (represented by the ability of triphenyltetrazolium chloride to be reduced), leaf photosynthetic rate, plant dry matter accumulation, and nitrogen accumulation at the flowering stage and fruit expanding stage; (2) to explore the compensatory influence of aeration for nitrogen topdressing reduction on tomato fruit yield and quality; and (3) to suggest the favorable aeration ratio and nitrogen topdressing level for tomato field cultivation with underground drip irrigation in arid and semi-arid areas.

## 2. Results

### 2.1. Influences of Aeration Ratios and Nitrogen Topdressing Levels on Root Vigor

The root absorption abilities of soil water and nutrients are closely related to the root vigor. During the tomato flowering period and fruit expanding period, the tomato root vigor for different aeration ratios and topdressing levels is shown in Table 1. The tomato root vigor for the aeration treatments was higher than that fir the non-aeration treatments; the A3F2 treatment obtained the maximum value of root vigor both at the flowering stage and fruit expanding stage, while the A0F3 treatment achieved the minimum root vigor. With the same nitrogen topdressing level, the A3 aeration ratio obtained the highest root vigor, while with same aeration proportion, the root vigor for the F2 nitrogen topdressing level was higher than that for the F1 and F3 nitrogen topdressing levels. Under non-aeration conditions, there was no significant difference in the root vigor for different treatments at the flowering stage. With the same topdressing level, the root vigor for aeration treatments was significantly higher than that for the non-aeration treatments during the fruit enlargement period. Additionally, the analysis of variance results (Table 1) showed that both the aeration ratio and nitrogen topdressing level exhibited highly significant (*p* < 0.01) effects on tomato root vigor at the flowering stage and fruit expanding stage. Though the interaction of the two factors had extremely significant influences on root vigor at the tomato fruit expanding stage, the two factors exhibited no significant effect on root vigor at the tomato flowering stage in 2020.

### 2.2. Effects of Aeration Ratios and Nitrogen Topdressing Levels on Leaf Net Photosynthetic Rate

**Figure 1:**
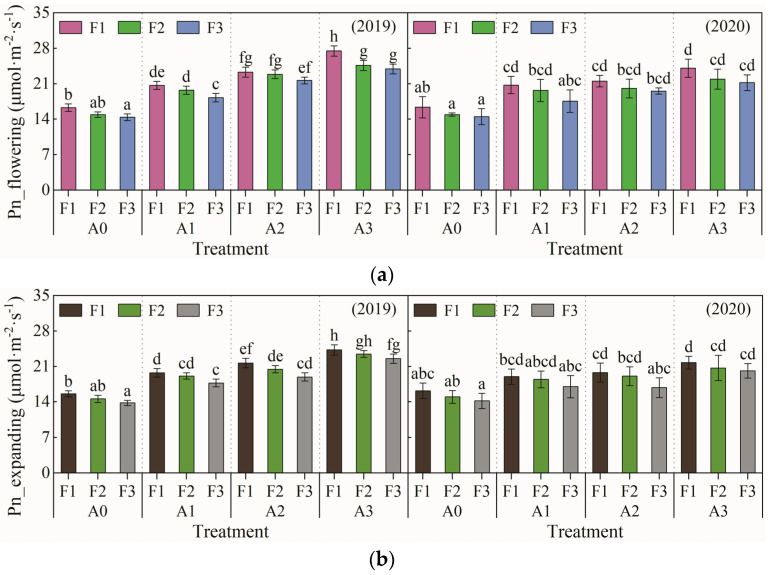
Leaf net photosynthetic rate at the tomato flowering stage and fruit expanding stage. (**a**) Leaf net photosynthetic rate at the tomato flowering stage (Pn_flowering); (**b**) leaf net photosynthetic rate at the tomato fruit expanding stage (Pn_expanding). Note: Different letters above the bars mean a significant difference according to the LSD test (*p* < 0.05).

As one of the most important metabolic processes in plants, photosynthesis is very sensitive to the contents of rootzone oxygen and nutrients. The tomato leaf net photosynthetic rate for treatments at the flowering stage and fruit enlargement stage are shown in Figure 1a,b. As shown in the significance analysis in Table 2, both at the flowering stage and the fruit expanding stage, the aeration ratio exerted a highly significant (*p* < 0.01) influence on the leaf net photosynthetic rate (Pn), while the two-factor interaction exhibited no significant effects (*p* > 0.05) on leaf Pn.

At both the flowering stage and fruit expanding stage of tomato plants, the Pn for aeration treatments was significantly higher than that for the non-aeration treatments. The A3F1 treatment and A0F3 treatment achieved the maximum and minimum Pn, respectively. With the same aeration percentage, leaf Pn decreased with the decline in nitrogen topdressing levels, and there was no significant difference in leaf Pn for treatments with the same nitrogen topdressing level. With the equivalent topdressing level, the leaf Pn increased with increasing aeration ratios. During the tomato flowering period, the leaf Pn for the A3F1 treatment increased by 46.6% compared with the A0F1 treatment; and the leaf Pn for the A2F2 treatment increased by 39.6% compared with the A0F2 treatment. The leaf Pn for the A2F2 treatment was 27.4% higher than the corresponding value for the A0F2 treatment. Furthermore, in terms of the same treatment, the leaf Pn at the flowering stage was higher than the corresponding value at the fruit enlargement stage. In particular, the variation amplitude in leaf Pn for the A2F3 treatment was 12.7%, which was higher than the values for the other treatments.

### 2.3. Impacts of Aeration Ratios and Nitrogen Topdressing Levels on Plant Dry Matter Accumulation

The significance analysis regarding the effects of nitrogen topdressing levels and aeration ratios on the dry matter accumulation of tomato plant tissues (root, stem, leaf, and fruit) is shown in Table 3. The influence of topdressing levels on the plant tissue dry matter accumulation at the flowering stage was extremely significant, and the effect on plant stem dry matter accumulation at the fruit enlargement stage was not significant (*p* > 0.05). The aeration ratio had significant effects on plant tissue dry matter accumulation during the flowering period and fruit enlargement period of tomato (*p* < 0.01). The interaction of the two factors showed significant influences on the dry matter accumulation of tomato roots and fruits at the flowering stage, and only showed significant effects on the stem dry matter accumulation at the fruit expansion stage (*p* < 0.05).

**Figure 2:**
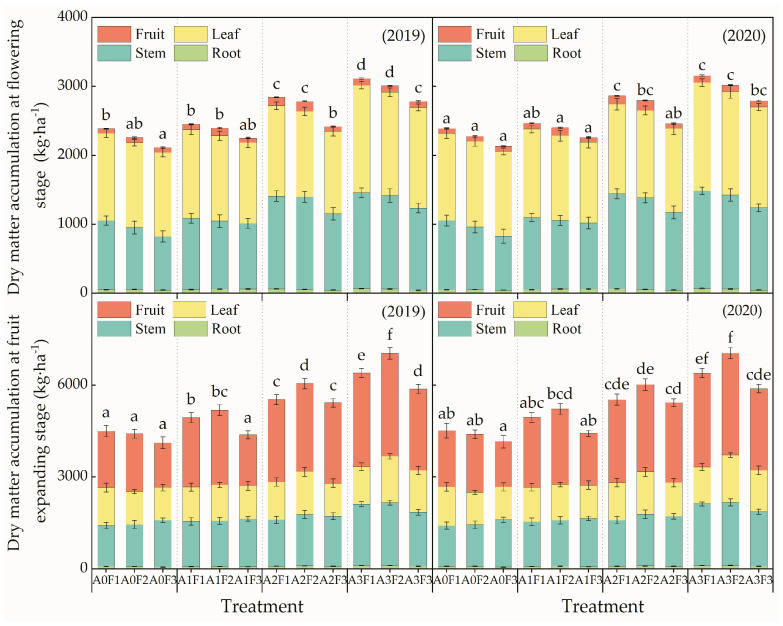
Dry matter accumulation of plant tissues at the flowering stage and fruit enlargement stage for different treatments. Note: Different letters above the bars indicate a significant difference according to the LSD test at *p* < 0.05.

At the flowering stage of tomato plants, the A3F1 treatment obtained the highest value of plant dry matter accumulation, while the A0F3 treatment obtained the lowest value (Figure 2). The dry matter accumulation for leaves (44.48–57.50% of the plants’ total dry matter accumulation) was larger than other tissues, followed by the stem dry matter accumulation (36.72–48.27%), and the dry matter accumulation for roots was the lowest.

With the same aeration ratio, the dry matter accumulation of stems, leaves, and total plant matter decreased with the decline in the nitrogen topdressing level, and no significant difference in plant dry matter accumulation was observed between the treatments with F1 and F2 nitrogen topdressing levels. Additionally, with aeration levels of A2 and A3, the dry matter amount for treatments with the F3 nitrogen topdressing level was significantly lower than that for treatments with the F1 and F2 nitrogen topdressing levels. With the same nitrogen topdressing level, although the effects of the A1 aeration ratio on plant dry matter accumulation was not significant, compared with non-aeration treatment, the plant dry matter accumulation increased significantly with the increasing aeration ratio in the range of 5–15%.

During the fruit enlargement period, the A3F2 treatment achieved the highest plant dry matter accumulation. The fruit dry matter accumulation (about 35.61–48.64% of the total dry matter accumulation of plants) was larger than that of other tissues, followed by the stem dry matter accumulation, and the root dry matter accumulation (about 1.47–1.89% of the total dry matter accumulation of plants) was smaller than that of other tissues. For the non-aeration treatments, although the plant dry matter accumulation decreased with a reduction in the topdressing level, there was no significant difference in plant dry matter accumulation corresponding to treatments with various nitrogen topdressing levels. With the same aeration ratio, the dry matter accumulation amount of the roots, stems, leaves, and fruits increased first and then decreased with the decrease in topdressing level. In addition, with the A2 and A3 aeration ratios, the plant dry matter accumulation corresponding to the F2 nitrogen topdressing level was significantly higher than that for the F1 and F3 nitrogen topdressing levels. With the same nitrogen topdressing level, the dry matter accumulation of stems, fruits, and plants increased with the increase in the aeration ratio.

In addition, the dry matter accumulation of roots, stems, and fruits at the tomato fruit enlargement stage was higher than that at the flowering stage. However, the dry matter accumulation of leaves at the fruit enlargement stage was lower than that at the flowering stage. Compared with the proportion of dry matter accumulation of roots, stems, leaves, and fruits in the plants’ total dry matter accumulation at the flowering stage, the corresponding proportion of roots, stems, and leaves at the fruit enlargement stage decreased, while the corresponding proportion of fruits increased by 32–45%.

### 2.4. Impacts of Aeration Ratios and Nitrogen Topdressing Levels on Plant Nitrogen Accumulation

The results of variance analysis (Table 4) showed that the nitrogen topdressing level showed significant effects on the nitrogen accumulation of roots, stems, and fruits. The aeration proportion exhibited significant effects on the nitrogen accumulation of plant tissues at the flowering stage and fruit enlargement stage, while the interaction of the two factors had no significant influence on plants’ nitrogen accumulation.

**Figure 3:**
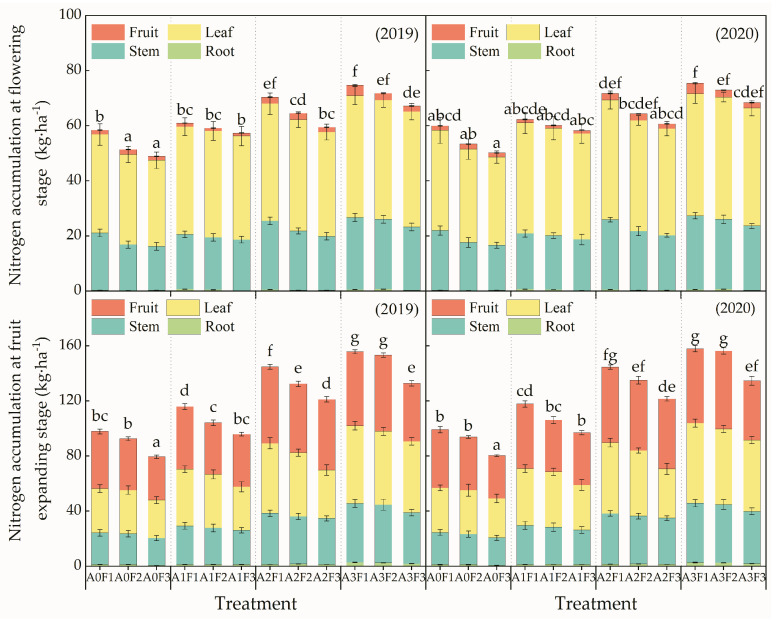
Nitrogen accumulation in tomato plant tissues at the flowering stage and the fruit enlargement stage for different treatments. Note: Different letters above the bars indicate a significant difference according to the LSD test at *p* < 0.05.

At the tomato flowering stage, the plant nitrogen was mainly accumulated in the stems and leaves, as shown in Figure 3. With the same nitrogen topdressing level, the plant total nitrogen accumulation increased with the increase in aeration ratios. Moreover, the nitrogen accumulation of stems and leaves increased with the increase in aeration ratios. In addition, the nitrogen accumulation amount for treatments with the A2 and A3 aeration ratios was significantly higher than that for the non-aeration treatments. With the same aeration ratio, there was no significant difference in plant nitrogen accumulation for treatments with the F2 and F3 nitrogen topdressing levels. The plant nitrogen accumulation decreased with the decline in nitrogen topdressing levels, and the leaves’ nitrogen accumulation amount decreased with the decline in topdressing levels.

During the fruit enlargement period of tomato plants, nitrogen was mainly accumulated in the fruits and leaves. The fruit nitrogen accumulation amount accounted for 31.75–42.44% of the total, while the root nitrogen accumulation only accounted for 0.81–1.82%. With the same nitrogen topdressing level, the plants’ and stems’ nitrogen accumulation increased significantly with the increase in the aeration ratio. With the same aeration level, the plant and stem nitrogen accumulation amount decreased with the decline in nitrogen topdressing levels. In addition, the leaf nitrogen accumulation decreased with the decline in nitrogen topdressing levels. However, the proportion of stem nitrogen increased with the decline in nitrogen topdressing levels. In terms of the tomato flowering stage and fruit expanding stage, the nitrogen accumulation amounts of tomato roots, fruits, and stems at the flowering stage were lower than those at the fruit expansion stage.

### 2.5. Impacts of Aeration Ratios and Nitrogen Topdressing Levels on Tomato Fruit Development

**Figure 4:**
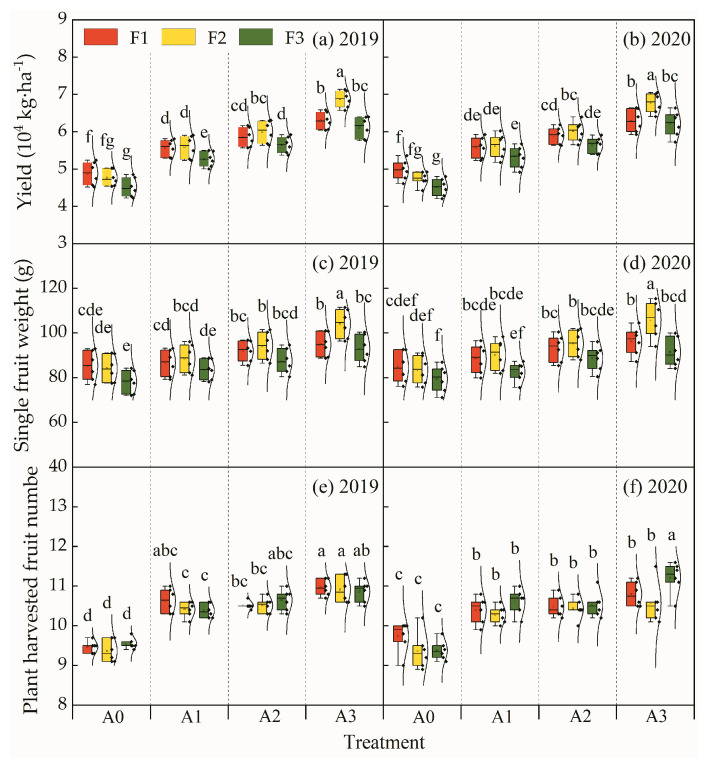
Tomato yield, single-fruit weight and number of harvested fruits per plant for treatments. Note: Different letters above the boxes mean a significant difference according to the LSD test at *p* < 0.05.

The single-fruit weight, tomato yield, and number of harvested fruits per plant for the treatments in 2019 and 2020 are shown in Figure 4. The A3F2 treatment obtained a higher single-fruit weight and tomato yield than the other treatments, being 104.2 g and 6.9 × 10^4^ kg·ha^−1^ in 2019 and 105.1 g and 6.8 × 10^4^ kg·ha^−1^ in 2020, respectively. Meanwhile, the A0F3 treatment obtained the lowest single-fruit weight and tomato yield, being 78.2 g and 4.5 × 10^4^ kg·ha^−1^ in 2019 and 78.9 g and 4.5 × 10^4^ kg·ha^−1^ in 2020, respectively. Moreover, the number of harvested fruits per plant for each treatment ranged between 9.4 and 11.2, and the values for the aeration treatments were significantly higher than those for the non-aeration treatments. The A3F1 treatment and A3F3 treatment provided the maximum value in 2019 and 2020, respectively. With the same topdressing level, both the single-fruit weight and tomato yield increased with increasing aeration proportions. In particular, the tomato yield for the A2 aeration ratio increased by approximately 20%, 26% and 25% compared to the non-aeration treatment at the F1, F2, and F3 nitrogen application levels, respectively. For the non-aeration treatments, both the single-fruit weight and tomato yield decreased with reducing topdressing levels. In terms of the same aeration ratio, the single-fruit weight and tomato yield for the F2 topdressing level were higher than those for the F1 and F3 topdressing levels. With same aeration ratio of A0, A1, or A2, the number of harvested fruits per plant for each treatment with designated nitrogen topdressing levels showed no significant difference. The analysis of variance results (Table 5) exhibited that the aeration ratio, the topdressing level and the two-factor interaction exhibited extremely significant (*p* < 0.01) effects on the single-fruit weight and tomato yield. Only the aeration ratio exhibited highly significant (*p* < 0.01) influences on the number of harvested fruits per plant.

### 2.6. Influences of Aeration Ratios and Nitrogen Topdressing Levels on Tomato Fruit Quality

**Figure 5:**
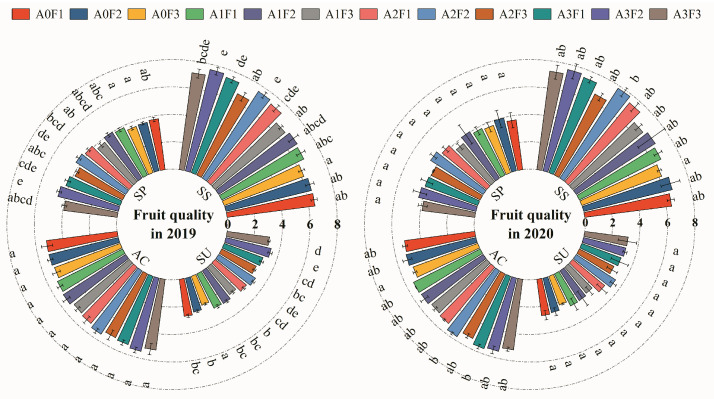
Tomato fruit quality for treatments in 2019 and 2020. SS, SU, AC, and SP represent the soluble solid content (10 mg·g^−1^), the soluble sugar content (10 mg·g^−1^), the organic acid content (mg·g^−1^), and the soluble protein content (mg·g^−1^). Note: Different letters following the values indicate a significant difference according to the LSD test at *p* < 0.05.

The A3F2 treatment and A0F3 treatment achieved the maximum and minimum contents of soluble solids, organic acid, soluble sugars, and soluble proteins in tomato fruits, respectively (Figure 5). No significant difference in organic acid content was observed for the treatments. The contents of soluble solids, soluble sugars, and soluble proteins of tomato fruit under aeration conditions were higher than those for the non-aeration treatments. The contents of soluble solids, soluble sugars, and soluble proteins decreased with reducing nitrogen topdressing levels under non-aeration conditions. With the same aeration ratio, the contents of soluble solids, soluble sugars, and soluble proteins for treatments with a nitrogen topdressing level of F2 were higher than the corresponding values for the nitrogen topdressing levels of F1 and F3. With the same topdressing level, the contents of soluble solids, soluble sugars, and soluble proteins increased with the increase in the aeration ratio. The extent of the increase in the soluble solid content and soluble sugar content for aeration treatments with an F2 nitrogen topdressing level was higher than the corresponding values for the non-aeration treatments with nitrogen topdressing levels of F1 and F3. Meanwhile, the extent of the increase in the soluble protein content for aeration treatments with an F3 nitrogen topdressing level was higher than that for the non-aeration treatments with nitrogen topdressing levels of F1 and F2.

The analysis of variance results (Table 6) indicated that both the aeration ratio and the nitrogen topdressing level exhibited highly significant (*p* < 0.01) influences on tomato fruit organic acid content, while no significant (*p* > 0.05) effects on soluble sugar content and soluble protein content were observed. Additionally, there was no significant (*p* > 0.05) impact of the two-factor interaction on fruit quality traits.

## 3. Discussion

### 3.1. The Response of Root Vigor to Aeration Ratios and Nitrogen Topdressing Levels

Aerated underground drip irrigation generally mixes water, fertilizer, and air synchronously and transports them to the crop rootzone. This is usually beneficial for alleviating the hypoxic conditions of the crop rootzone [3,5], conducive to promoting the aerobic respiration of crop roots [7,19], and favorable for improving the soil nutrient availability and soil nutrient utilization efficiency by crop roots. With aeration irrigation, the scientific reduction in the topdressing amount of chemical fertilizer can not only contribute to reducing the risk of farmland non-point source pollution, but also meet the fertilizer supply requirements for vigorous crop growth [16,23,24,25]. The present two-year field experiment showed that the aeration ratio exhibited highly significant effects on root vigor during the flowering period and fruit expanding period. This demonstrates that aeration is closely related to tomato root vigor. With the same aeration ratio, as the nitrogen topdressing amount reduced by 25%, the root vigor correspondingly increased by 5.4–10.7% at the tomato flowering stage and 6.8–29.9% at the fruit expanding stage. With the same topdressing level, the root vigor corresponding to an aeration ratio of 10% increased by 10.4–24.5% at the flowering stage and 13.3–103.9% at the fruit enlargement stage. In addition, the root vigor for the aeration treatments was higher than that for the non-aeration treatments at both the tomato flowering and fruit expanding stages. This result is in line with the results of Yang et al. (2019) and Baram et al. (2022) [9,26], which may be due to the fact that aeration results in the improvement of the rootzone soil microenvironment [9], as well as the increase in plants’ roots’ metabolism level [13].

### 3.2. The Response of Leaf Net Photosynthetic Rate to Aeration Ratios and Nitrogen Topdressing Levels

The results of significance analysis showed that the aeration ratio exhibits highly significant effects on leaf Pn at the tomato flowering stage and fruit expanding stage. Lowering the nitrogen topdressing level was observed to be detrimental to the improvement of leaf Pn. This result may be due to the nutrient deficiency at a low nitrogen topdressing level, which is not conducive to the roots’ growth and development. With the same nitrogen topdressing level, the leaf Pn showed an increasing trend with the increase in the aeration ratio—the leaf Pn for treatments with an aeration ratio of 10% increased by 37–54% compared with the non-aeration treatment. These results may be attributed to the ventilation with aeration during irrigation, which alleviates the soil hypoxic stress caused by the underground drip irrigation. Furthermore, soil moisture and nutrient absorption from the rootzone by crops was facilitated, and the photosynthesis rate was improved [27]. With the same aeration ratio, leaf Pn decreased with the reducing topdressing levels at the flowering stage and fruit expanding stage. Moreover, the leaf Pn decreased by 2–11% as the nitrogen topdressing amount declined by 25%. In addition, the A3F1 and A0F3 treatments achieved the maximum value and minimum value of leaf Pn, respectively. These results regarding the leaf net photosynthetic rate are consistent with those in existing research [8,28,29]. Li et al. (2019) found that rhizosphere soil aeration promoted chlorophyll accumulation in tomato leaves [8]. Parveen et al. (2021) showed that the chlorophyll content and wheat leaves stomatal conductance increased after aeration, and the leaf Pn and transpiration rate increased accordingly [29].

### 3.3. The Response of Tomato Drymatter to Aeration Ratios and Nitrogen Topdressing Levels

Aerated irrigation can effectively increase the soil oxygen content, alleviate the low-soil-oxygen stress in the rootzone of plants, and promote the dry matter accumulation of plants [4,16]. At the tomato flowering stage and fruit enlargement stage, the aeration ratios and nitrogen topdressing levels have very significant effects on the dry matter accumulation of tomato plants. This indicates that the aeration ratios and nitrogen topdressing levels are closely related to the tomato dry matter accumulation. With the same topdressing level, the plants’ dry matter accumulation amount increased with the increasing aeration ratio. This may be due to the increase in ventilation with underground drip irrigation, which effectively alleviates the soil hypoxic stress caused by buried drip irrigation. This is more conducive to the respiration of plant roots and the absorption of soil moisture and nutrient elements, thus promoting the synthesis of photosynthetic products and plant dry matter accumulation. The present experiment showed that, with the same aeration ratio, as the nitrogen topdressing level decreased from 80 kg·ha^−1^ to 40 kg·ha^−1^, the proportion of root dry matter in plants’ dry matter decreased with the decrease in the topdressing level at the flowering and fruit setting stages. Meanwhile, the percentage first increased and then decreased with the decrease in the topdressing level at the fruit expansion stage. This result shows that the appropriate reduction in the topdressing level was beneficial for tomato plant roots’ growth and development. In addition, the A3F2 treatment obtained the highest dry matter accumulation during the fruit expansion period, which indicated that with a high aeration ratio (A3), appropriately reducing the topdressing level was beneficial to plants’ dry matter accumulation. Aeration irrigation effectively improved the ventilation of crop roots, which in turn exhibited positive effects on tomato plants’ growth and development. The dry matter accumulation of the roots, stems, and fruits at the fruit enlargement stage was higher than that at the flowering stage, while the leaves’ dry matter accumulation at the fruit enlargement stage was lower than that at the flowering stage. This implies that the leaves’ dry matter accumulation rate is less than the leaves’ dry matter consumption rate, from the flowering stage to the fruit expansion stage, and the dry matter accumulated by plants is mainly distributed in the roots, stems and fruits.

### 3.4. The Response of Tomato Nitrogen Distribution to Aeration Ratios and Nitrogen Topdressing Levels

Some studies have shown that nitrogen plays an important role in the growth of stems and leaves and fruit development and is closely related to tomato yield [30]. The results of this experiment show that tomato plants’ nitrogen accumulation is closely related to aeration ratios and topdressing levels. With the same aeration ratio, the plants’ nitrogen accumulation decreased with the decrease in nitrogen topdressing level, which indicates that reducing nitrogen topdressing level is not conducive to plant nitrogen accumulation. The aerated subsurface drip irrigation technology usually mixes water, fertilizer, and gas and synchronously transports them to tomato rootzone. This is beneficial for alleviating the hypoxic conditions in the crop rootzone [5], increasing the the antioxidant enzyme activity in the roots, promoting the aerobic respiration of soil microorganisms in the crop rootzone [9], directly promoting the soil bacterial and fungal communities, and improving the effectiveness of crop roots utilizing soil nutrients [2]. In this study, the plants’ nitrogen accumulation increased with the increase in the aeration ratio under the same topdressing level, which indicated that aeration in the crops’ rootzone was beneficial to promoting plant nutrients accumulation [31]. In this experiment, nitrogen was mainly accumulated in the fruits and leaves during the tomato fruit expansion period, which was consistent with the results of Wu et al. [32], who found that the leaves’ nitrogen content is higher than that in the roots, stems, and fruits.

### 3.5. The Response of Tomato Fruit Development to Aeration Ratios and Nitrogen Topdressing Levels

Soil hypoxia usually inhibits the utilization of soil water and soil nutrients by the plant root system, which is not conducive to plant photosynthesis, and often exhibits adverse effects on various physiological activities during the plant growth period, which may result in crop yield reduction and fruit quality decline [7,23]. The present experiments found that both the aeration ratio and the nitrogen topdressing level had extremely significant effects on the single-fruit weight and tomato yield. With the same nitrogen topdressing level, both the single-fruit weight and tomato yield increased with the increasing aeration rate; moreover, the tomato yield increased by 20–26% when the aeration ratio reached 10% compared to 0%. This result indicates that the increase in the aeration ratio with underground drip irrigation was conducive to an increase in the single-fruit weight and tomato yield. That result was in accordance with the results of Pendergast et al. (2013) and Bagatur et al. (2014), as they found that rootzone aeration can accelerate crop growth and development, increase crop yield, increase single-fruit weight, and improve crop quality [3,33]. Moreover, previous studies have indicated that the increase in tomato yield with aeration irrigation is mainly attributed to the increase in single-fruit weight [34]. In addition, with the same aeration ratio, the tomato yield increased by 0.4–9.1% when the nitrogen topdressing amount decreased by 25%; the single-fruit weight and tomato yield for the A3F2 treatment were significantly higher than those for the A3F1 treatment and A3F3 treatment. These results indicate that both an excessive nitrogen topdressing rate and an insufficient nitrogen topdressing rate exhibit adverse effects on crop production [35]. Additionally, aeration in the underground drip irrigation system is conducive to significantly increasing the number of ripe tomato fruits per plant. This may be due to the fact that aeration in the underground drip irrigation system contributes to an increase in the tomato fruit setting rate.

The tomato fruit organic acid content was significantly affected by aeration ratios and nitrogen topdressing levels, while the soluble sugar content and soluble protein content were not significantly affected by the aeration proportions and nitrogen topdressing levels. The ratios of soluble sugar content to organic acid content for designed treatments ranged from 7 to 8.3. This implies that the tomato taste quality was excellent, since the optimal sugar–acid ratio for tomato fruit is in the range of 7–10 [36]. With the same nitrogen topdressing level, the contents of fruit soluble solids, soluble sugars, and soluble proteins increased with the increasing aeration ratios; specifically, the contents of soluble solids and organic acids of tomato fruit increased by 3–19% and 2–9%, respectively. These results indicate that aeration in the underground drip irrigation system is beneficial to improving tomato fruits’ nutritional quality (soluble solids, soluble protein content) and flavor (soluble sugar). These results are partially similar to the findings of Zhang et al. (2022) [16], as Zhang et al. (2022) indicated that rootzone aeration could significantly increase tomato fruits’ soluble solid content, soluble sugar concentration, organic acid content, soluble protein concentration, and vitamin C content [16]. Additionally, the present study found that the F3 nitrogen topdressing level tended to be more conducive to significantly improving the tomato nutritional quality, while the F2 nitrogen topdressing level was more conducive to significantly improving the tomato fruit taste quality.

### 3.6. The Optimization of the Aeration Ratio and Topdressing Level for Underground Drip Irrigation during the Tomato Growth Period

In terms of root vigor improvement, the aeration ratio and nitrogen topdressing level corresponding to the A3F2 treatment were better than those of the other treatments, followed by the A2F2 treatment. There was no significant difference in root vigor between the A3F2 treatment and the A2F2 treatment at the tomato flowering stage, but the root vigor for the A3F2 treatment was significantly higher than that for the A2F2 treatment at the fruit expanding stage. The root vigor for the A3F2 treatment was about 26% and 130% higher than that for the A0F1 treatment at the flowering stage and the fruit enlargement stage, respectively. To improve the tomato leaf Pn, the A3F1 treatment was better than the A3F2 treatment, whereas no significant difference in the leaf Pn between the A3F1 treatment and A3F2 treatment was found. Compared with the A3F1 treatment, the leaf Pn for the A3F2 treatment decreased by less than 10% at the flowering stage and less than 5% at the fruit enlargement stage. Although the dry matter for the A2F2 treatment was lower than the maximum value obtained by the A3F1 treatment and the A3F2 treatment at the flowering and fruit enlargement stages, respectively, the difference between the A2F2 and A3F1 treatments at the flowering stage was less than 11%, while the difference between the A2F2 and A3F2 treatments at the fruit enlargement stage was less than 15%. In addition, the nitrogen accumulation amount for the A2F2 and A3F1 treatments was less than 14% at the flowering stage and lower than 15% at the fruit expansion stage, respectively. In terms of improving tomato single-fruit weight and fruit yield, the A3F2 treatment was more appropriate than the other treatments, followed by the A2F2 treatment. Moreover, the single-fruit weight and tomato yield for the A3F2 treatment increased by 38–41% and 22–24%, respectively, compared with the A0F1 treatment. There were significant differences in the single-fruit weight and tomato yield between the A3F2 and A2F2 treatments. To improve the contents of soluble solids, soluble sugars, organic acids, and soluble proteins of tomato fruit, the A3F2 treatment was better than the other treatments in 2019, while the A2F2 treatment was more appropriate in 2020. In both 2019 and 2020, there was no significant difference in the same characteristic values of tomato fruit between the A3F2 treatment and the A2F2 treatment—the differences in the soluble solid content, soluble sugar content, organic acid content, and soluble protein content were less than 3%, 6%, 4%, and 7%, respectively.

With the designed experimental conditions, taking the reduction in the nitrogen topdressing amount during the tomato growth period, the increase in the tomato yield and the improvement of fruit quality into consideration, the aeration ratio and nitrogen topdressing level corresponding to the A3F2 treatment were suggested in this study. However, limited by the experimental conditions, the nitrogen topdressing levels and aeration ratios were lower. The application of more precise aeration ratios and nitrogen topdressing levels for tomato underground drip irrigation remains to be studied.

## 4. Materials and Methods

### 4.1. Experimental Site and Climate

**Figure 6:**
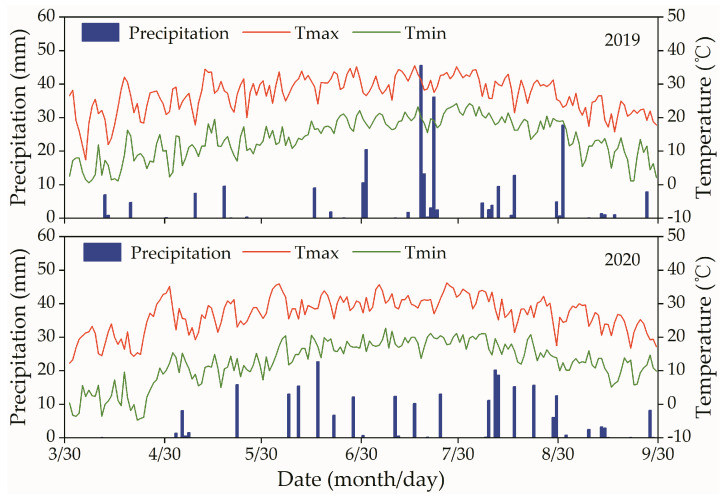
The daily precipitation, maximum temperature (Tmax), and minimum temperature (Tmin) at a 2 m height in the experimental site in 2019 and 2020.

The two-year field experiment was conducted from 2019 to 2020 in the Yinbei Irrigation District of Ningxia Hui Autonomous Region, China (longitude: 106.58° E, latitude: 38.85° N, 1096 m a.s.l.). The experimental area experiences a mesothermal arid continental climate characterized by sparse rainfall (average annual rainfall of 183.4 mm) and high evaporation (average multi-year evaporation of 1702 mm), and the average annual sunshine duration and total solar radiation are 3010 h and 5600 MJ·m^−1^, respectively. Moreover, the climate features an average relative humidity of 45% and an average annual temperature of 8 °C. The upper 40 cm of soil and the 40–100 cm deep layer of soil can be as classified sandy loam and silty clay, respectively. The average buried depth of groundwater in the experimental site is 2.2 m, while the groundwater salinity is 1.26 g·L^−1^. An automatic weather station (YM-03A, Handan Yimeng Electronics Co., Ltd., Handan, China) was installed 28 m away from the experimental field, and the meteorological parameters, including daily rainfall, photosynthetically active radiation, extreme temperature, wind speed and relative humidity, were recorded at intervals of 60 min. The daily precipitation, maximum temperature, and minimum temperature from 1 April to 30 September in 2019 and 2020 are shown in Figure 6.

### 4.2. Experimental Design

The tomato hybrid variety “Fenda No. 1”, a traditional variety suitable for outdoor planting in spring or summer in temperate arid climate areas, was used for the field experiments. This prevalent hybrid variety was selected by the Ningxia Academy of Agriculture and Forestry Sciences in China, and the ripe fruits are characterized by an oblate shape and pink color. According to the traditional nitrogen application rate (20 kg·ha^−1^ for base fertilization and 80 kg·ha^−1^ for topdressing) for tomato cultivation with underground drip irrigation in the experimental area, the main plots of the field experiments in open air comprised four aeration ratios (proportions of air volume to mixed liquid volume), and three nitrogen topdressing levels were applied to sub-plots. The four aeration ratios were 0 (A0), 5% (A1), 10% (A2), and 15% (A3), while the three nitrogen topdressing levels were 80 kg·ha^−1^ (F1), 60 kg·ha^−1^ (F2), and 40 kg·ha^−1^ (F3). The twelve treatments were replicated three times with 12 plants in each row and about 67 plants in each column, and thus about 800 plants per replicate.

Each experimental plot for a treatment covered an area of 156 m^2^ (20 m in length and 7.8 m in width). Each treatment was equipped with an independent drip irrigation system. Infiltration tubes with an emitter spacing of 0.3 m and a flow rate of 1.38 L·h^−1^ (Shandong Yangtze River Water Saving Irrigation Science and Technology Co., Ltd., Jinan, China) were placed between the plants and buried 30 cm underground on 25 April 2019 and 20 April 2020, respectively. The spacing between the two adjacent infiltration pipes was 130 cm. Meanwhile, the base fertilizer, comprising urea (N, 46%) at rate of 45 kg·ha^−1^, triple superphosphate (P_2_O_5_, 50%) at a rate of 100 kg·ha^−1^, and potassium sulfate (K_2_O, 46%) at a rate of 120 kg·ha^−1^, was spread uniformly in the experimental field.

**Figure 7:**
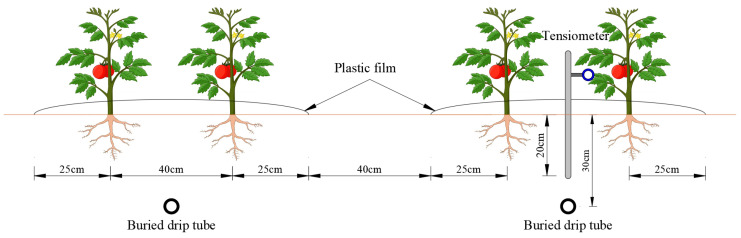
Schematic diagram of tomato planting.

Tensiometers were buried 10 cm above the infiltration tube on 28 April 2019 and 25 April 2020, respectively, for the purpose of guiding the drip irrigation. Each experimental treatment was equipped with one water meter, one functioning tensiometer, six raised beds, and six subsurface irrigation tubes. On 3 May 2019 and 1 May 2020, healthy tomato seedlings were transplanted into the beds of the experimental field with a row spacing of 40 cm and a plant spacing of 30 cm (Figure 7), and the designed planting density was about 5 plants per square meter. Each treatment was drip-irrigated once to ensure the survival rate of transplanted seedlings with local shallow groundwater (single irrigation quota of 30 mm) on 4 May 2019 and 2 May 2020.

Tensiometer readings were observed three times (08:00, 13:00, and 18:00) every day after the tomato seedings were transplanted. As soon as the records reached −20 kPa (the soil moisture at 20 cm depth below the drip irrigation tube was approximately 80% of the field moisture capacity), drip irrigation was applied at 18:00 on the same day with a single irrigation quota of 20 mm. Drip irrigation events were terminated when the tomato fruit entered the red ripening period to prevent the occurrence of blossom end rot. 

**Figure 8:**
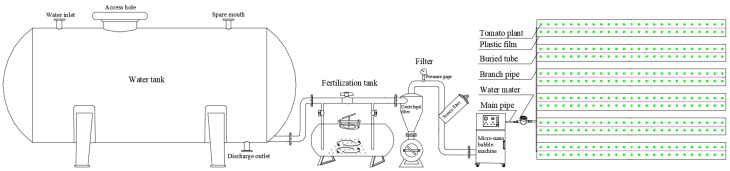
Schematic diagram of the test device.

**Figure 9:**
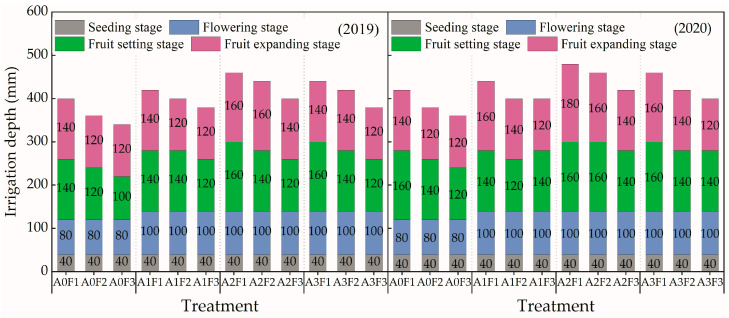
The irrigation depth for the treatments in 2019 and 2020. Note: The marked data on each bar represent the accumulated irrigation depth in the corresponding stage.

As shown in Figure 8, the drip tubes of the same treatment were connected to the micro-nano bubble machine to transport the water–air mixture to the plots. According to the designed proportions, irrigation water was aerated via the micro-nano bubble machine (ZJC-NM-02, Shanghai Zhongjing Environmental Protection Technology Co., Ltd., Shanghai, China) by controlling the working pressure of the micro-nano bubble machine (0, 0.03, 0.06, 0.11 MPa) to control the gas ratio (0, 5%, 10%, 15%) and mixing the bubbles (average diameter of 74 nm) into the local shallow groundwater. Then, the water–air mixture was delivered to each treatment through the underground drip irrigation system. Three micro-nano bubble machines were used for the experiments, as each machine had a water outflow of 2 m^3^·h^−1^ and served 12 plots. The detailed accumulated irrigation depth for treatments at the seeding, flowering, fruit setting and fruit expanding stages are shown in Figure 9, and the total irrigation depth for treatments during tomato growth period ranged from 340 mm to 460 mm. Additionally, the topdressing times were the first, second, fourth, and sixth ear flowering stages of tomato, respectively, and the proportion of the nitrogen amounts for the four topdressing times was 1:1:1:1. Other agronomic measures were kept the same as in local conventional tomato cultivation.

### 4.3. Observation and Equipment

#### 4.3.1. Soil Physical and Chemical Parameters

Undisturbed soil samples were collected at 20 cm intervals 0–100 cm downward from the surface on 29 April 2019 and 26 April 2020, respectively, for the determination of soil physical and chemical characteristics. Soil moisture and bulk density were measured with the gravimetric method [37], and the soil texture was analyzed using a laser particle size analyzer (Master-sizer 3000, Malvern Instruments Ltd., Malvern, UK). The contents of soil organic matter and total nitrogen were determined using the potassium dichromate oxidation external heating method [38] and Kjeldahl method [39], respectively, while the contents of available nitrogen, available phosphorus, and available potassium in the soil layers were measured with the alkaline dissolved diffusion method [40], the molybdenum-antimony colorimetric method, and the flame photometer method, respectively [41]. Soil samples for the determination of pH and dissolved salt content were air-dried first, ground and sieved through a 2 mm sieve to prepare a solution with a soil–water ratio of 1:5, then centrifuged and oscillated (rotational speed r = 5000 turns per minute) to test the EC_1:5_ with a conductivity meter (DDS-308A, Shanghai Precision Scientific Instrument Co., Ltd., Shanghai, China). Moreover, the soil pH was determined using a pH meter (PHS-3C, Shanghai Precision Scientific Instrument Co., Ltd., Shanghai, China). Some of the physical and chemical characteristics in the 0–40 cm and 40–100 cm soil layers are shown in Table 7.

The buried depth of the local groundwater was observed using a self-recording hydrometer, which was arranged in the groundwater observation well (5 m in depth and located 10 m away from the experimental field). According to the observation results of the previous experiments, the salinity of the shallow groundwater over the course of the year was approximately 0.5 g·L^−1^. According to the sampling examination results, the pH, COD, and suspended solid content of the local shallow groundwater were 7.5, 38.1 mg·L^−1^, and 6.7 mg·L^−1^, respectively. Moreover, the contents of dissolved salt and the main salt ions in the local shallow groundwater are shown in Table 8.

#### 4.3.2. Root Vigor and Leaf Net Photosynthetic Rate

Three consistent tomato plants were selected in each treatment at the flowering stage (2 July 2019 and 6 July 2020) and fruit expanding stage (28 July 2019 and 30 July 2020), and the roots were taken out from the soil in a circle of 0.4 m around the main root at a depth of 0.6 m. Root samples were cleaned with fresh water, air-dried, and weighed using a 1/1000 scale. Root vigor was determined using the “2, 3, 5-triphenyltetrazolium chloride (TTC)” method according to Xue et al. (2023) [42]. After the root samples were cleaned, the spectrophotometer (ND1000, Thermo Fisher Scientific Inc., Waltham, MA, USA) and some chemical reagents (ethyl acetate, sodium hydrosulfite, 1% TTC solution, 0.1 M phosphate buffer with pH at 7.5, and 2N sulfuric acid) were used to measure the TTC reduction amount, and then the root vigor (*Rv*) was calculated according to the following equation:

(1) $$Rv=\frac{RA_{TTC}}{W_{Root}\times T}$$

where Rv represents the root vigor (g·g^−1^·h^−1^); $RA_{TTC}$ represents the TTC reduction amount (g); $W_{Root}$ represents the measured root weight (g); and T represents the duration of the experiment (h).

The leaf Pn was measured by the CIRAS-3 portable photosynthetic measurement system (PP SYSTEMS, Amesbury, MA, USA) at the flowering stage and fruit expanding stage, at 60 days and 78 days after planting, respectively. The photosynthetically active radiation (PAR) from the LED light source was set as 1400 μmol·m^−2^‧s^−1^, while the other conditions included 70% relative humidity, a 500 μmol‧s^−1^ flow rate, and a CO_2_ concentration at 400 μmol·mol^−1^ through a CO_2_ buffer bottle during the measurement. The temperature of the leaf varied from 28 to 30 °C during the entire period of gas-exchange measurements. Six leaves were randomly selected to measure the Pn from 9:00 to 11:00 on sunny days for each treatment.

#### 4.3.3. Plant Dry Matter Accumulation and Nitrogen Accumulation

At the flowering stage and fruit expansion stage, three consecutive tomato plants were randomly selected from each experimental plot and the roots were removed from the rootzone. Plant samples were divided into four parts: roots, stems, leaves, and fruits. After de-enzyming at 105 °C for 0.5 h, the tissue samples were dried to a constant weight at 75 °C to determine the dry matter. Then, the dried tissue samples were milled through a 0.5 mm sieve and digested by a H_2_SO_4_-H_2_O_2_ solution, and the Kjeldahl method [39] was applied for measuring the plant tissues’ nitrogen content.

The nitrogen accumulation amount of plants was estimated using the following equations:

(2) $$U_{N}=\sum_{i=1}^{4}U_{Ni}$$

(3) $$U_{Ni}=\rho \times c_{Ni}\times T_{mi}$$

where *U_N_* represents the nitrogen accumulation amount of plants, kg·ha^−1^; *U_Ni_* represents the nitrogen accumulation amount of the *i*th tissue of plants, kg·ha^−1^; *C_Ni_* represents the nitrogen content of the *i*th tissue of a single plant, %; *T_mi_* represents the dry matter weight of the *i*th tissue, kilograms per plant; ρ represents the number of tomato plants per hectare; and *i* = 1, 2, 3, 4 represent the roots, stem, leaves, and fruits of the tomato plant, respectively.

#### 4.3.4. Tomato Yield

Tomatoes were harvested at 7-day intervals from 15 August to 18 September 2019 and from 17 August to 20 September 2020. During the tomato harvest period, the number of red-ripe fruits for each plant was recorded, and seven fruits for each treatment were randomly selected to determine the single-fruit weight. The tomato yield was estimated with the actual fruit production and planting area for each treatment.

#### 4.3.5. Tomato Fruit Quality

After the tomato harvest, three uniform-maturity fruits were selected from each treatment and homogenized for quality determination. Fruit soluble solid content was determined with the refractometric method [43]. Moreover, the soluble sugar content and organic acid content were determined with the Lane–Eynon method [43] and the high-performance liquid chromatography method [43], respectively. Additionally, the fruit soluble protein content was determined with the Coomassie brilliant blue G-205 dye method [44].

### 4.4. Statistical Analysis

The least significant difference (LSD) test was performed with SPSS 24.0 (SPSS Inc., Chicago, IL, USA) based on the data to compare the differences among treatments at a significance level of 5%. The root vigor, leaf net photosynthetic rate, dry matter accumulation, nitrogen accumulation, and fruit characteristics were analyzed via two-way analysis of variance with the factors of the aeration ratio (A), nitrogen topdressing level (F) and the interaction of A × F. The figures were drawn using OriginPro 2021 (OriginLab Corporation, Northampton, MA, USA).

## 5. Conclusions

With the experimental designed conditions, the aeration ratios exhibited extremely significant effects on root vigor, leaf net photosynthetic rate, plant dry matter, plant nitrogen accumulation, single-fruit weight, tomato yield, and organic acid content. Meanwhile, the interaction of aeration ratio and nitrogen topdressing level showed no significant influences on the leaf net photosynthetic rate, leaf dry matter, stem nitrogen accumulation, plant dry matter, plant nitrogen accumulation, and fruit quality characteristics.

The increase in aeration ratio in the range of 0–15% was beneficial for improving tomato root vigor (3–104%) and leaf photosynthetic rate (14–63%), favorable for promoting the accumulation of plant dry matter (3–59%) and plant nitrogen (2–70%), and conducive to increasing single-fruit weight (1–26%) and tomato yield (12–44%) and improving fruit quality. The tomato leaf net photosynthetic rate, leaf nitrogen accumulation, and plant nitrogen accumulation declined with the decrease in the nitrogen topdressing level in the range of 0–50%. Importantly, with aeration conditions, appropriately reducing the traditional nitrogen topdressing level by 25% was beneficial for increasing tomato root vigor (5–31%), favorable for promoting the dry matter accumulation of roots, stems, leaves, and fruits at the fruit expansion stage, increases the single-fruit weight (2–10%) and tomato yield (0.5–9%), and improves fruit soluble solid content (2–5%) and soluble sugar content (4–9%). When the aeration ratio exceeded 10%, increasing the aeration ratio exhibited a significant influence on tomato plant dry matter accumulation, while it had no significant effects on the single-fruit weight, tomato yield, and fruit quality. Moreover, the stem nitrogen content proportion in plants at the tomato fruit enlargement stage increased with the decline in the nitrogen topdressing level, while the fruit soluble solid content and soluble sugar content increased first and then decreased with the decrease in nitrogen topdressing level. In terms of leaf photosynthetic rate improvement, plant dry matter accumulation, tomato yield increase, fruit soluble solids accumulation, tomato soluble sugar formation, and fruit organic acid build-up, the positive effects of increasing the aeration ratio by 5% was greater than the negative influences of reducing the nitrogen topdressing level by 25%. To reduce the nitrogen topdressing application amount, promote plant growth, and achieve a considerable tomato yield, the appropriate nitrogen topdressing amount for tomato plants with underground drip irrigation was 60 kg·ha^−1^ in Yinbei Irrigation District of Ningxia, and the corresponding proper aeration ratio was 10%.

## Figures and Tables

**Table 1:** Effects of different levels of aeration and nitrogen topdressing on root vigor at the flowering stage and fruit expanding stage in 2019 and 2020. Separately for each source of variation and within each column, means followed by a common letter are not significantly different according to the LSD test (*p* < 0.05).

Source of Variation		2019		2020	
Aeration	Nitrogen Topdressing	Flowering Stage	Fruit Expanding Stage	Flowering Stage	Fruit Expanding Stage
A0	F1	1.31 ± 0.039 f	0.36 ± 0.076 ef	1.33 ± 0.043 gh	0.38 ± 0.061 e
A0	F2	1.38 ± 0.057 ef	0.43 ± 0.029 de	1.40 ± 0.035 efg	0.44 ± 0.051 de
A0	F3	1.29 ± 0.019 f	0.34 ± 0.053 f	1.28 ± 0.014 h	0.35 ± 0.067 e
A1	F1	1.38 ± 0.051 ef	0.52 ± 0.034 c	1.38 ± 0.071 fgh	0.53 ± 0.055 c
A1	F2	1.52 ± 0.049 cd	0.48 ± 0.041 cd	1.54 ± 0.044 bcd	0.49 ± 0.025 cd
A1	F3	1.49 ± 0.053 d	0.40 ± 0.005 ef	1.49 ± 0.041 de	0.41 ± 0.051 de
A2	F1	1.47 ± 0.065 de	0.63 ± 0.039 b	1.46 ± 0.043 def	0.64 ± 0.048 b
A2	F2	1.62 ± 0.054 ab	0.68 ± 0.029 b	1.64 ± 0.064 ab	0.68 ± 0.020 b
A2	F3	1.42 ± 0.068 de	0.54 ± 0.049 c	1.45 ± 0.085 def	0.55 ± 0.036 c
A3	F1	1.61 ± 0.026 bc	0.67 ± 0.037 b	1.62 ± 0.048 abc	0.68 ± 0.059 b
A3	F2	1.71 ± 0.084 a	0.87 ± 0.030 a	1.71 ± 0.063 a	0.89 ± 0.016 a
A3	F3	1.51 ± 0.065 cd	0.62 ± 0.029 b	1.53 ± 0.071 b	0.63 ± 0.031 b
Significance level					
Aeration ratio		**	**	**	**
Nitrogen topdressing level		**	**	**	**
Aeration ratio × Nitrogen topdressing level		*	**	ns	**

Note: ns, *, and ** indicate no significant difference (*p* > 0.05), significant difference (*p* < 0.05), and extremely significant difference (*p* < 0.01) according to the two-way ANOVA, respectively.

**Table 2:** Significance levels of treatments’ impacts on net photosynthetic rates.

Source of Variation	2019		2020	
	Flowering Stage	Fruit Expanding Stage	Flowering Stage	Fruit Expanding Stage
Aeration ratio	**	**	**	**
Nitrogen topdressing level	**	*	ns	ns
Aeration ratio × Nitrogen topdressing level	ns	ns	ns	ns

Note: ns, *, and ** indicate no significant difference (*p* > 0.05), significant difference (*p* < 0.05), and extremely significant difference (*p* < 0.01) according to the two-way ANOVA, respectively.

**Table 3:** Significance analysis of dry matter accumulation in plant tissues.

Source of Variation	Flowering Stage					Fruit Expanding Stage				
	Root	Stem	Leaf	Fruit	Plant	Root	Stem	Leaf	Fruit	Plant
Aeration ratio	*	**	**	**	**	**	**	**	**	**
Nitrogen topdressing level	**	**	**	**	**	**	ns	*	**	**
Aeration ratio × Nitrogen topdressing level	**	ns	ns	**	ns	ns	*	ns	ns	ns

Note: ns, *, and ** indicate no significant difference (*p* > 0.05), significant difference (*p* < 0.05), and extremely significant difference (*p* < 0.01) according to the two-way ANOVA, respectively.

**Table 4:** Significant analysis of nitrogen accumulation in tomato plants.

Source of Variation	Flowering Stage					Fruit Expanding Stage				
	Root	Stem	Leaf	Fruit	Plant	Root	Stem	Leaf	Fruit	Plant
Aeration ratio	**	**	**	**	**	**	**	**	**	**
Nitrogen topdressing level	**	**	ns	**	**	**	**	**	**	**
Aeration ratio × Nitrogen topdressing level	**	ns	ns	**	ns	**	ns	*	**	ns

Note: ns, *, and ** indicate no significant difference (*p* > 0.05), significant difference (*p* < 0.05), and extremely significant difference (*p* < 0.01) according to the two-way ANOVA, respectively.

**Table 5:** Significance levels of treatments’ impacts on tomato yield, single-fruit weight and number of harvested fruits per plant.

Source of Variation	2019			2020		
	Single-Fruit Weight	Tomato Yield	Fruit Number	Single-Fruit Weight	Tomato Yield	Fruit Number
Aeration ratio	**	**	**	**	**	**
Nitrogen topdressing level	**	**	ns	**	**	*
Aeration ratio × Nitrogen topdressing level	**	**	ns	**	**	ns

Note: ns, *, and ** indicate no significant difference (*p* > 0.05), significant difference (*p* < 0.05), and extremely significant difference (*p* < 0.01) according to the two-way ANOVA, respectively.

**Table 6:** Significance levels of treatments’ effects on tomato fruit quality traits in 2019 and 2020.

Source of Variation	2019				2020			
	SS	SU	AC	SP	SS	SU	AC	SP
Aeration ratio	ns	ns	**	ns	*	ns	**	ns
Topdressing level	ns	ns	**	ns	*	ns	**	ns
Aeration ratio × Topdressing level	ns	ns	ns	ns	ns	ns	ns	ns

Note: SS, AC, SU, and SP represent the soluble solid content, organic acid content, soluble sugar content and soluble protein content; ns, *, and ** indicate no significant difference (*p* > 0.05), significant difference (*p* < 0.05), and extremely significant difference (*p* < 0.01) according to the two-way ANOVA, respectively.

**Table 7:** Soil physical and chemical characteristics in the experimental field.

Soil Depth (cm)	Texture	Bulk Density (g·cm^−3^)	Field Capacity (%)	Porosity (%)	pH	Total Salt Content (g·kg^−1^)	Available Nitrogen (mg·kg^−1^)	Available Phosphorus (mg·kg^−1^)	Available Potassium (mg·kg^−1^)
0–40	Sandy loam	1.39	19.72	40.27	8.54	1.47	51.10	8.20	254.50
40–100	Silty clay	1.33	17.37	38.33	7.81	1.22	47.97	3.35	123.50

**Table 8:** The contents of dissolved salt and the main salt ions in the local shallow groundwater.

Dissolved Salt (mg·L^−1^)	Ca^2+^ (mg·L^−1^)	Mg^2+^ (mg·L^−1^)	K^+^ (mg·L^−1^)	Na^+^ (mg·L^−1^)	HCO_3_^−^ (mg·L^−1^)	CO_3_^2−^ (mg·L^−1^)	Cl^−^ (mg·L^−1^)	SO_4_^2−^ (mg·L^−1^)
1259.4	155.5	96.0	136.5	215.4	182.4	0.0	275.1	198.5

## Data Availability

The data presented in this study are available upon request from the corresponding author.
